# Supplementary material for: Development platform for artificial pancreas algorithms
Source: PLoS One. 2020 Dec 17;15(12):e0243139. doi: 10.1371/journal.pone.0243139 (PMC7746189; doi:10.1371/journal.pone.0243139)
Supplement: S1 Material — (PDF) [file pone.0243139.s001.pdf]

S1 Material

Connecting a user algorithm to the Development Platform

- Figure S1: Connecting a T1DM closed-loop algorithm written in Matlab code to the Development Platform though the Nexus Communicator Plugin.
- Figure S2: Connecting a T1DM closed-loop algorithm written in Java code to the Development Platform though the Nexus Communicator Plugin.

Interacting with Development Platform

- Figure S3: Desktop Login interface to the Development Platform.
- Figure S4: Development Platform welcome screen.
- Figure S5: Selecting a population of virtual patients.
- Figure S6: Algorithm Performance Report assessing the expected clinical outcomes of a 24-hour CSII treatment emulating the CSII treatment of CLASS03.
- Figure S7: Virtual Patient-13 response graph during the CSII treatment.
- Figure S8: Virtual Patient-13 response graph during the single-hormone treatment.
- Figure S9: Algorithm Performance Report assessing the expected clinical outcomes of a 24-hour dual-hormone treatment emulating the dual-hormone treatment of CLASS03.
- Figure S10: Virtual Patient-13 response graph during the dual-hormone treatment.

Tables

- Table S1. Baseline characteristics of study participants
- Table S2. Comparisons of dual-hormone artificial pancreas, single-hormone artificial pancreas, and conventional insulin pump therapy for the overall Simulation study period (0800 h to 0800 h)
- Table S3. Overnight (2300 h to 0800 h) comparisons between Simulated and Real Experiments using clinical data collected in the CLASS03 randomized trial involving the dual-hormone artificial pancreas, single-hormone artificial pancreas, and conventional insulin pump therapy arms.

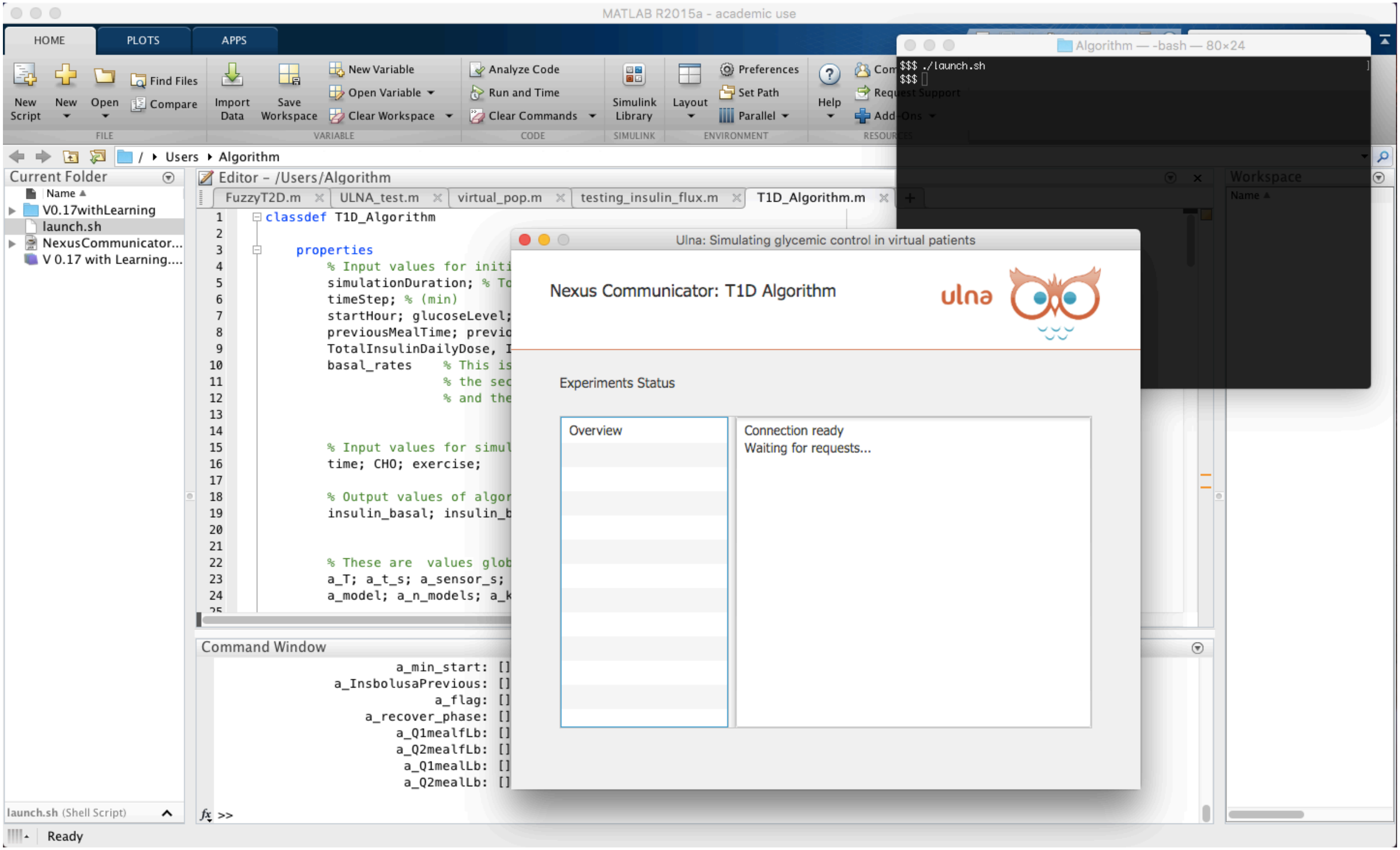

Figure S1: Connecting a T1DM closed-loop algorithm written in Matlab code to the Development Platform though the Nexus Communicator Plugin. The Matlab code implements two functions of an interface class to connect to the Platform. The plugin uses the two functions to communicate insulin / glucagon dosages to the Platform and receives glucose responses and meal input. A user launches a shell script to execute the connection protocol to the Platform and run an instance of their Matlab algorithm.

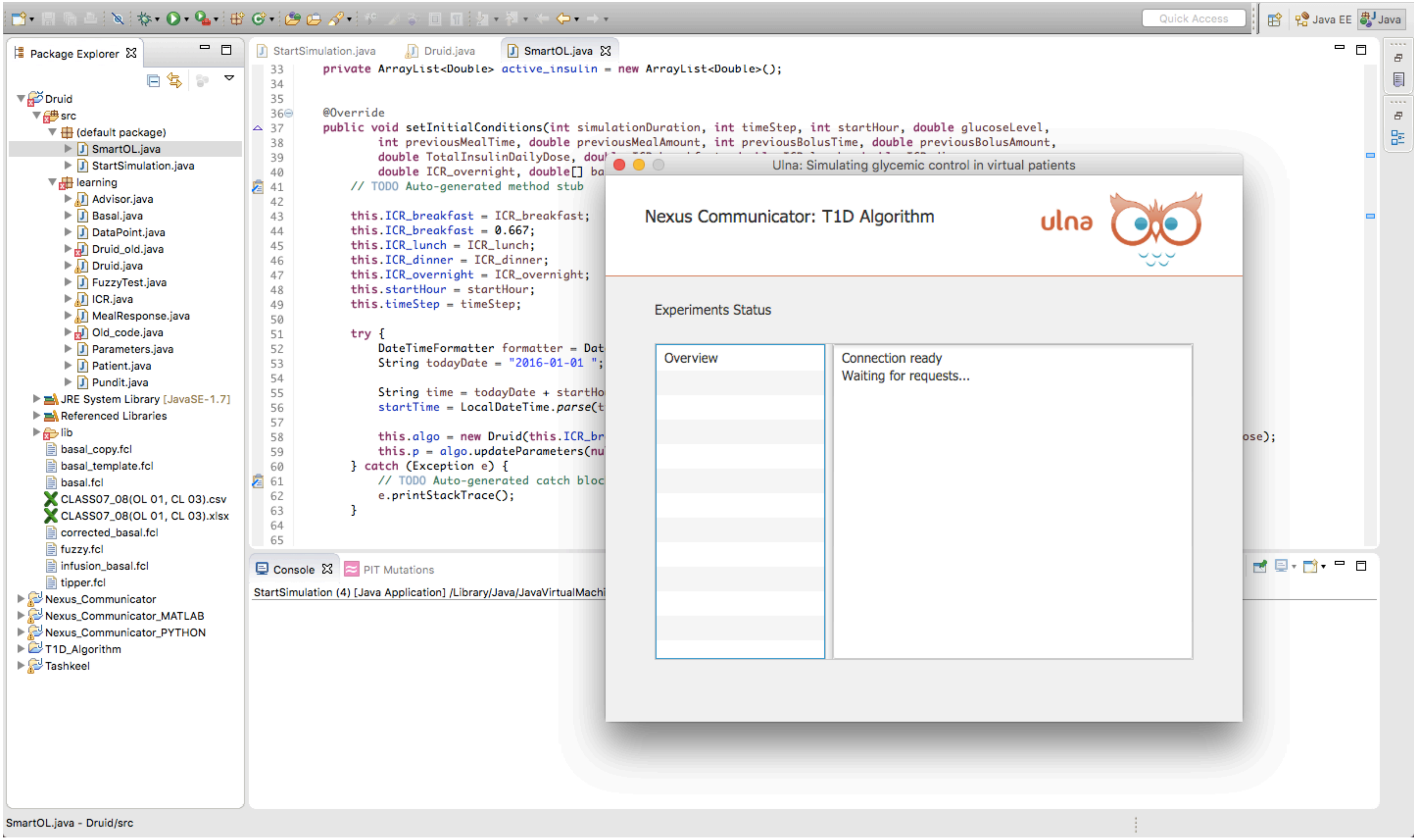

Figure S2: Connecting a T1DM closed-loop algorithm written in Java code to the Development Platform though the Nexus Communicator Plugin. The Java code implements an interface class to connect to the Platform. The plugin uses the two methods to communicate insulin / glucagon dosages to the Platform and receives glucose responses and meal input. A user imports the NexusCommunicator_JAVA.jar library into their code and automatically connects to the Platform upon running their Java algorithm.

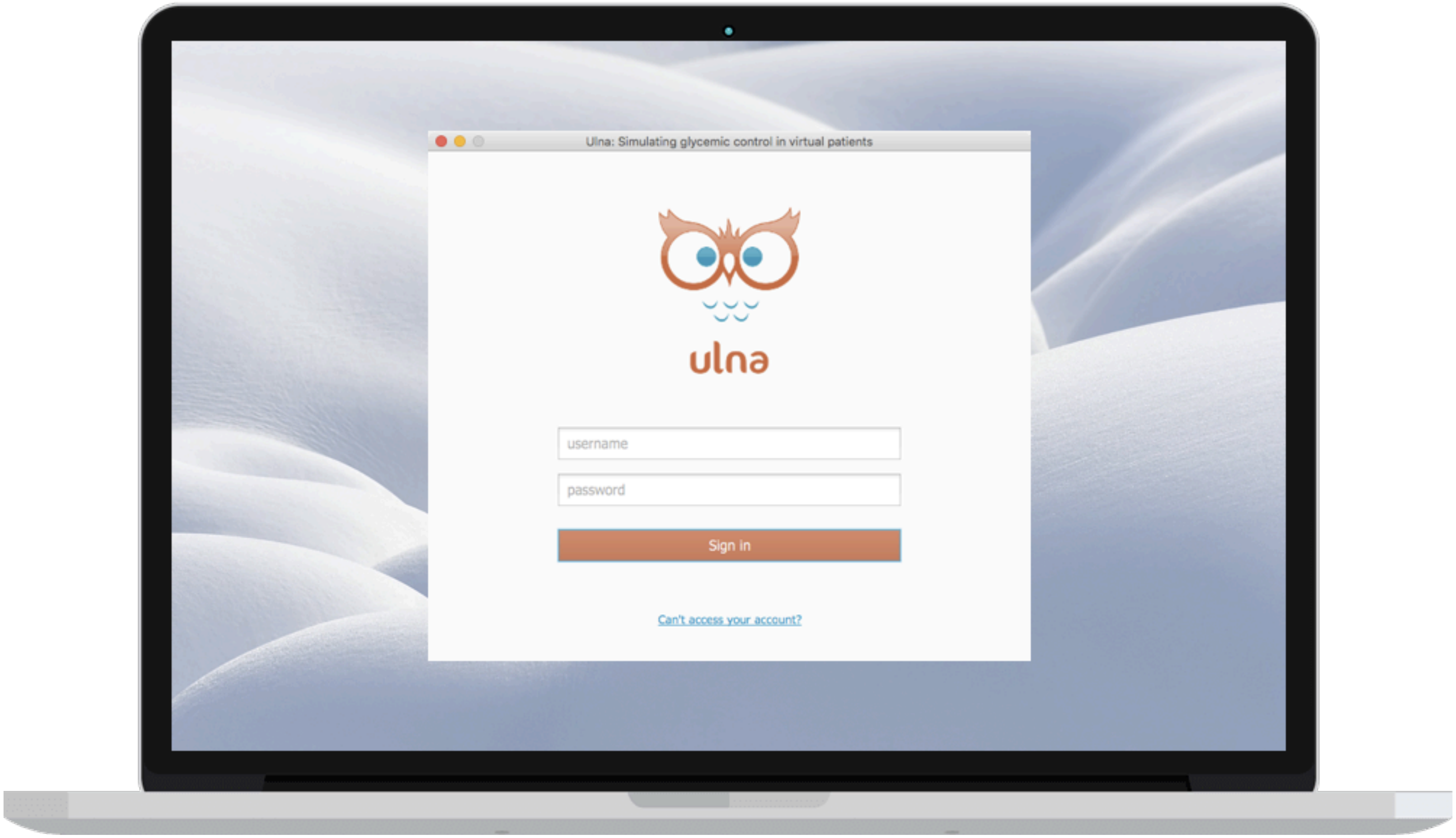

Figure S3: Desktop login interface to the Development Platform. Current versions of the application run on both Windows and MacOS systems. Algorithm developers can download the application from http://t1dclinic.com/ulna.php

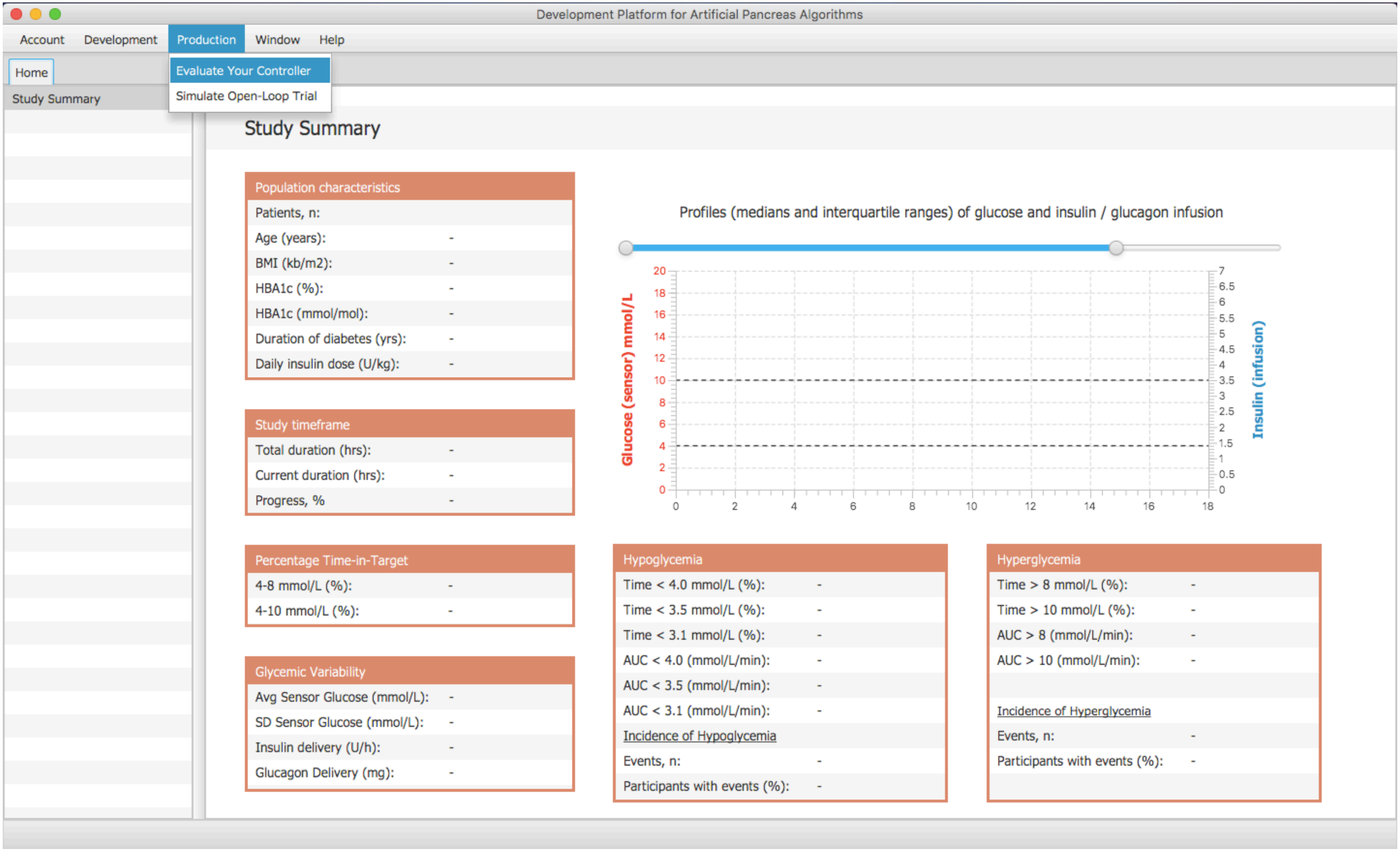

Figure S4: Development Platform welcome screen. A user can select "Evaluate Your Controller" from the Production panel to launch a virtual clinical trial.

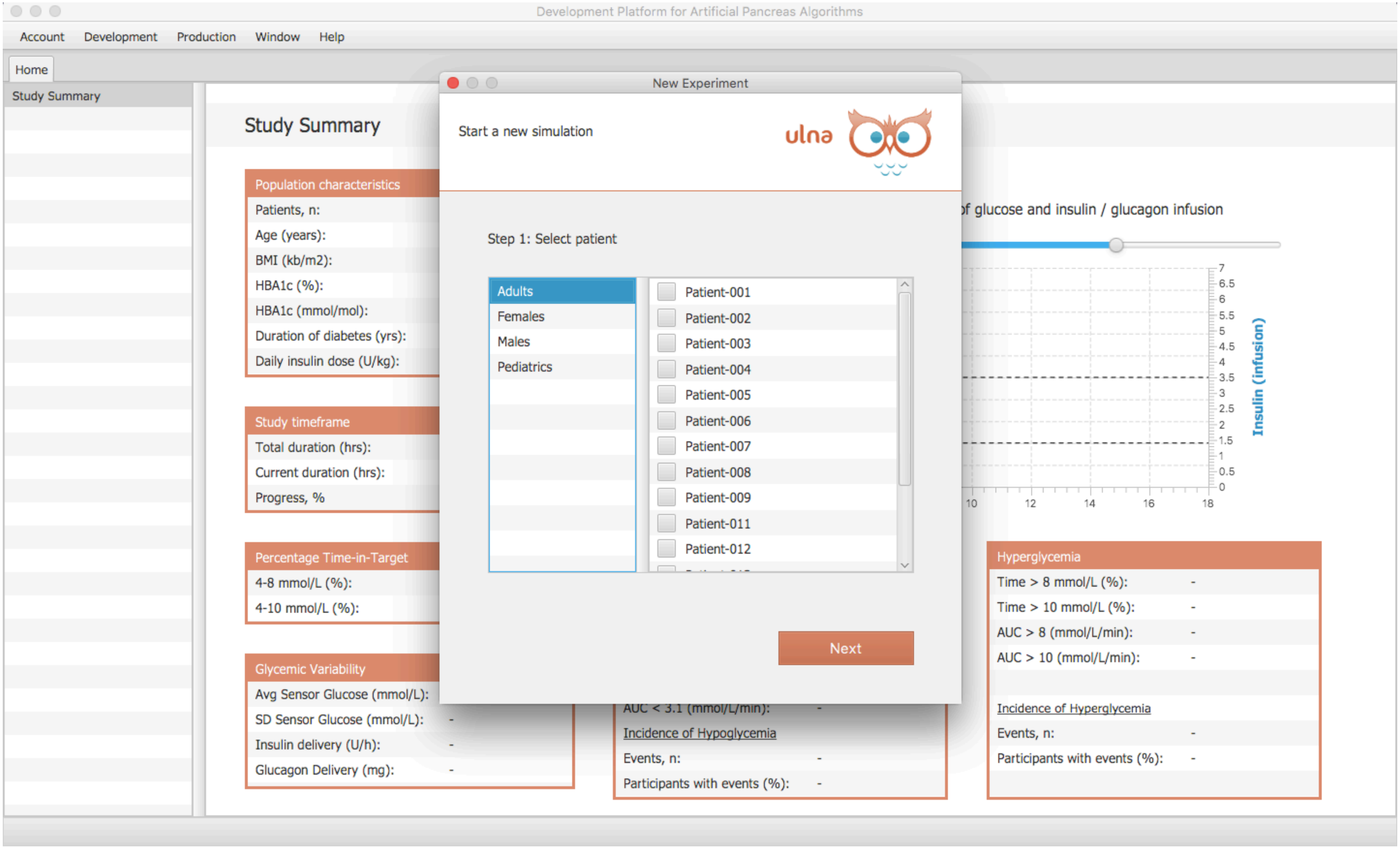

Figure S5: Selecting a population of virtual patients. The first step in launching a virtual clinical trial is to select the virtual subjects that will participate in the trial. The current version of the platform includes 15 virtual subjects. Upon addition of more subjects to the platform's database on the cloud, the panel will automatically show the new patients.

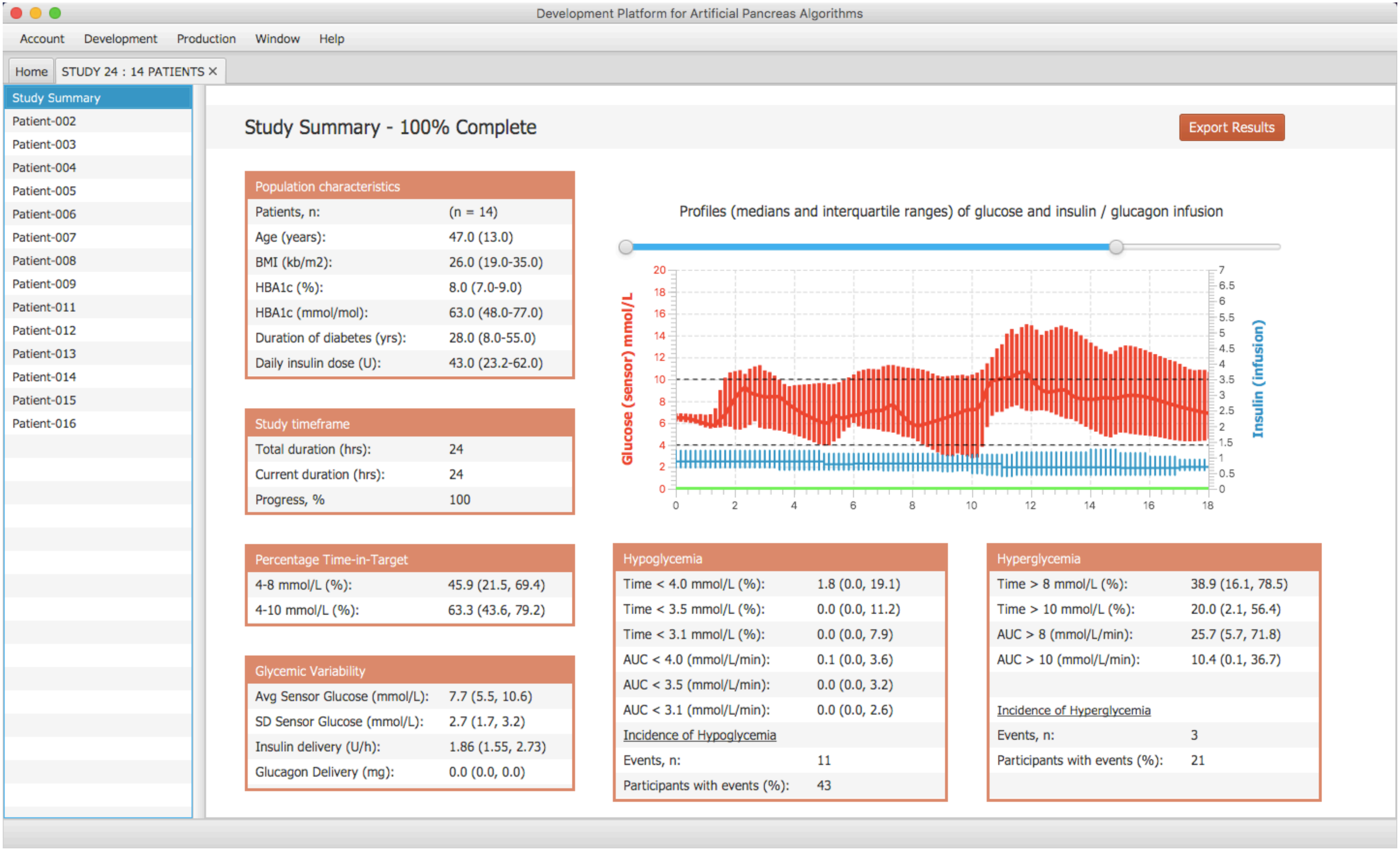

Figure S6: Algorithm Performance Report assessing the expected clinical outcomes of a 24-hour CSII treatment emulating the CSII treatment of CLASS03. 15 virtual subjects were given 4 meals over a 24-hour period (8 am to 8 am). The first meal was given at 8 am (median 59 g of carbohydrates, IQR (40-60)), the second meal at noon (70 g, 70-75), the third meal at 5 pm (95 g, 81-100), and a snack at 9 pm (20 g, 20-30).

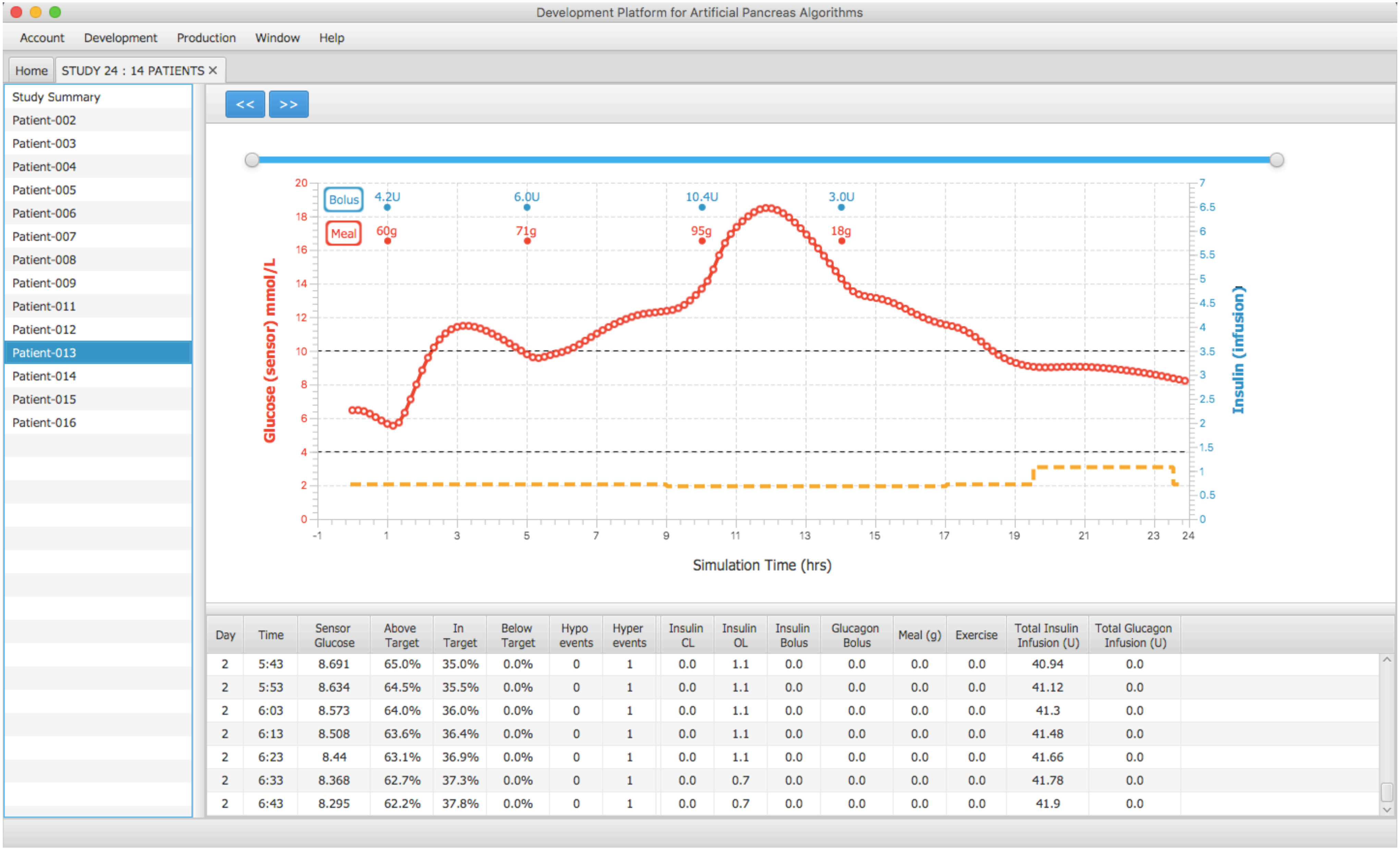

Day	Time	Sensor Glucose	Above Target	In Target	Below Target	Hypo events	Hyper events	Insulin CL	Insulin OL	Insulin Bolus	Glucagon Bolus	Meal (g)	Exercise	Total Insulin Infusion (U)	Total Glucagon Infusion (U)
2	5:43	8.691	65.0%	35.0%	0.0%	0	1	0.0	1.1	0.0	0.0	0.0	0.0	40.94	0.0
2	5:53	8.634	64.5%	35.5%	0.0%	0	1	0.0	1.1	0.0	0.0	0.0	0.0	41.12	0.0
2	6:03	8.573	64.0%	36.0%	0.0%	0	1	0.0	1.1	0.0	0.0	0.0	0.0	41.3	0.0
2	6:13	8.508	63.6%	36.4%	0.0%	0	1	0.0	1.1	0.0	0.0	0.0	0.0	41.48	0.0
2	6:23	8.44	63.1%	36.9%	0.0%	0	1	0.0	1.1	0.0	0.0	0.0	0.0	41.66	0.0
2	6:33	8.368	62.7%	37.3%	0.0%	0	1	0.0	0.7	0.0	0.0	0.0	0.0	41.78	0.0
2	6:43	8.295	62.2%	37.8%	0.0%	0	1	0.0	0.7	0.0	0.0	0.0	0.0	41.9	0.0

Figure S7: Virtual Patient-13 response graph during the CSII treatment.

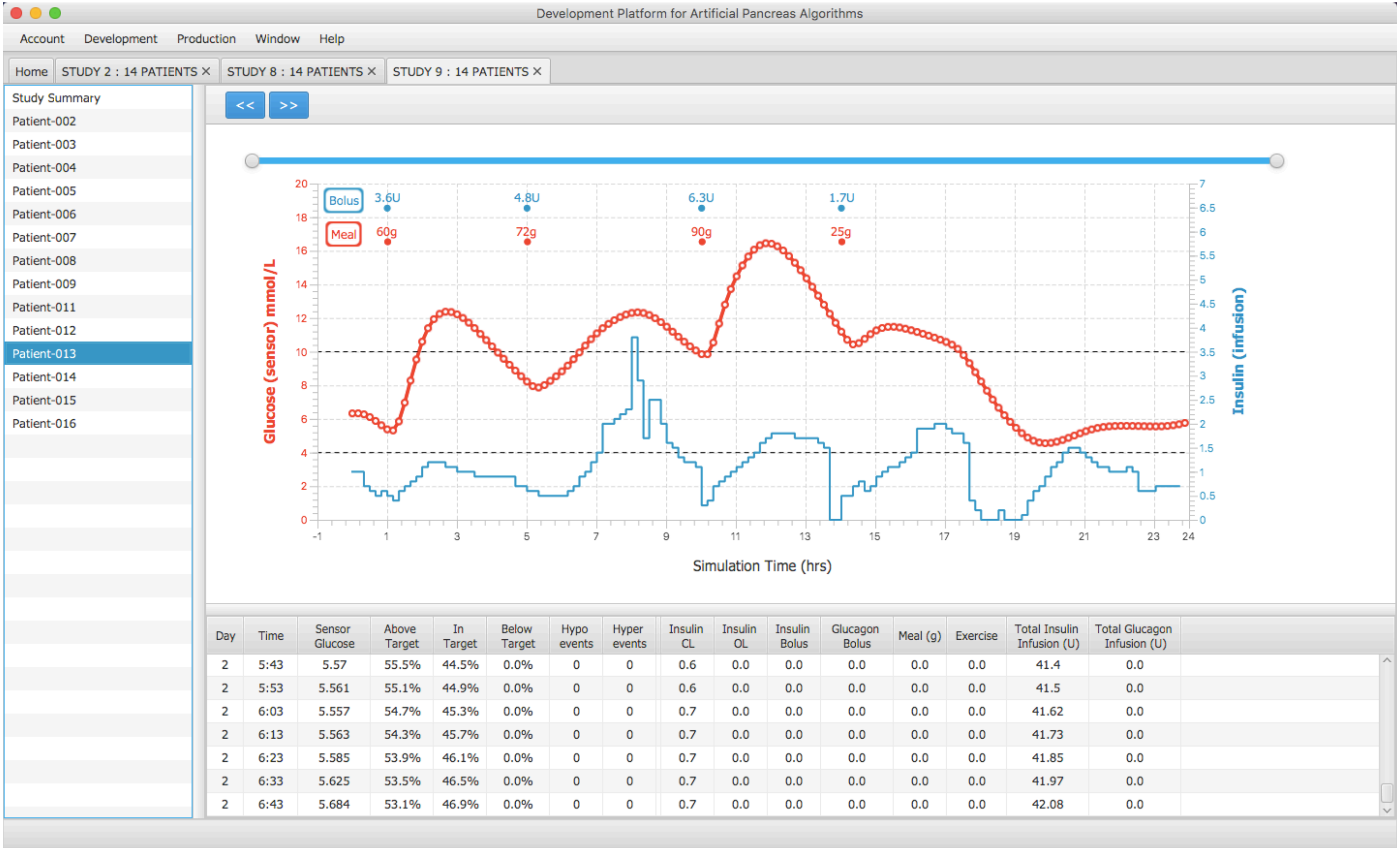

Day	Time	Sensor Glucose	Above Target	In Target	Below Target	Hypo events	Hyper events	Insulin CL	Insulin OL	Insulin Bolus	Glucagon Bolus	Meal (g)	Exercise	Total Insulin Infusion (U)	Total Glucagon Infusion (U)
2	5:43	5.57	55.5%	44.5%	0.0%	0	0	0.6	0.0	0.0	0.0	0.0	0.0	41.4	0.0
2	5:53	5.561	55.1%	44.9%	0.0%	0	0	0.6	0.0	0.0	0.0	0.0	0.0	41.5	0.0
2	6:03	5.557	54.7%	45.3%	0.0%	0	0	0.7	0.0	0.0	0.0	0.0	0.0	41.62	0.0
2	6:13	5.563	54.3%	45.7%	0.0%	0	0	0.7	0.0	0.0	0.0	0.0	0.0	41.73	0.0
2	6:23	5.585	53.9%	46.1%	0.0%	0	0	0.7	0.0	0.0	0.0	0.0	0.0	41.85	0.0
2	6:33	5.625	53.5%	46.5%	0.0%	0	0	0.7	0.0	0.0	0.0	0.0	0.0	41.97	0.0
2	6:43	5.684	53.1%	46.9%	0.0%	0	0	0.7	0.0	0.0	0.0	0.0	0.0	42.08	0.0

Figure S8: Virtual Patient-13 response graph during the single-hormone treatment.

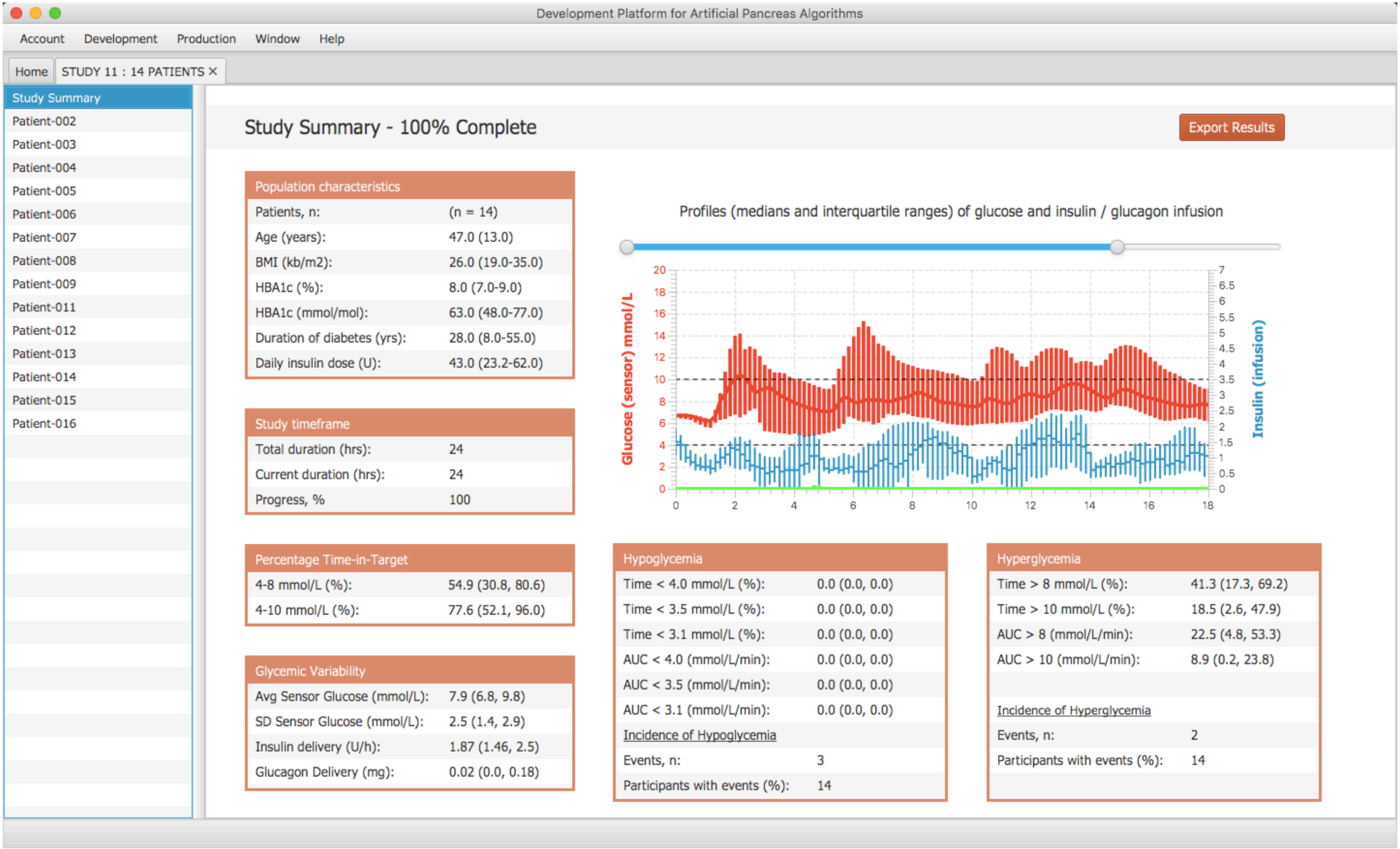

Figure S9: Algorithm Performance Report assessing the expected clinical outcomes of a 24-hour dual-hormone treatment emulating the dual-hormone treatment of CLASS03. 15 virtual subjects were given 4 meals over a 24-hour period (8 am to 8 am). The first meal was given at 8 am (median 59 g of carbohydrates, IQR (40-60)), the second meal at noon (70 g, 70-75), the third meal at 5 pm (95 g, 81-100), and a snack at 9 pm (20 g, 20-30).

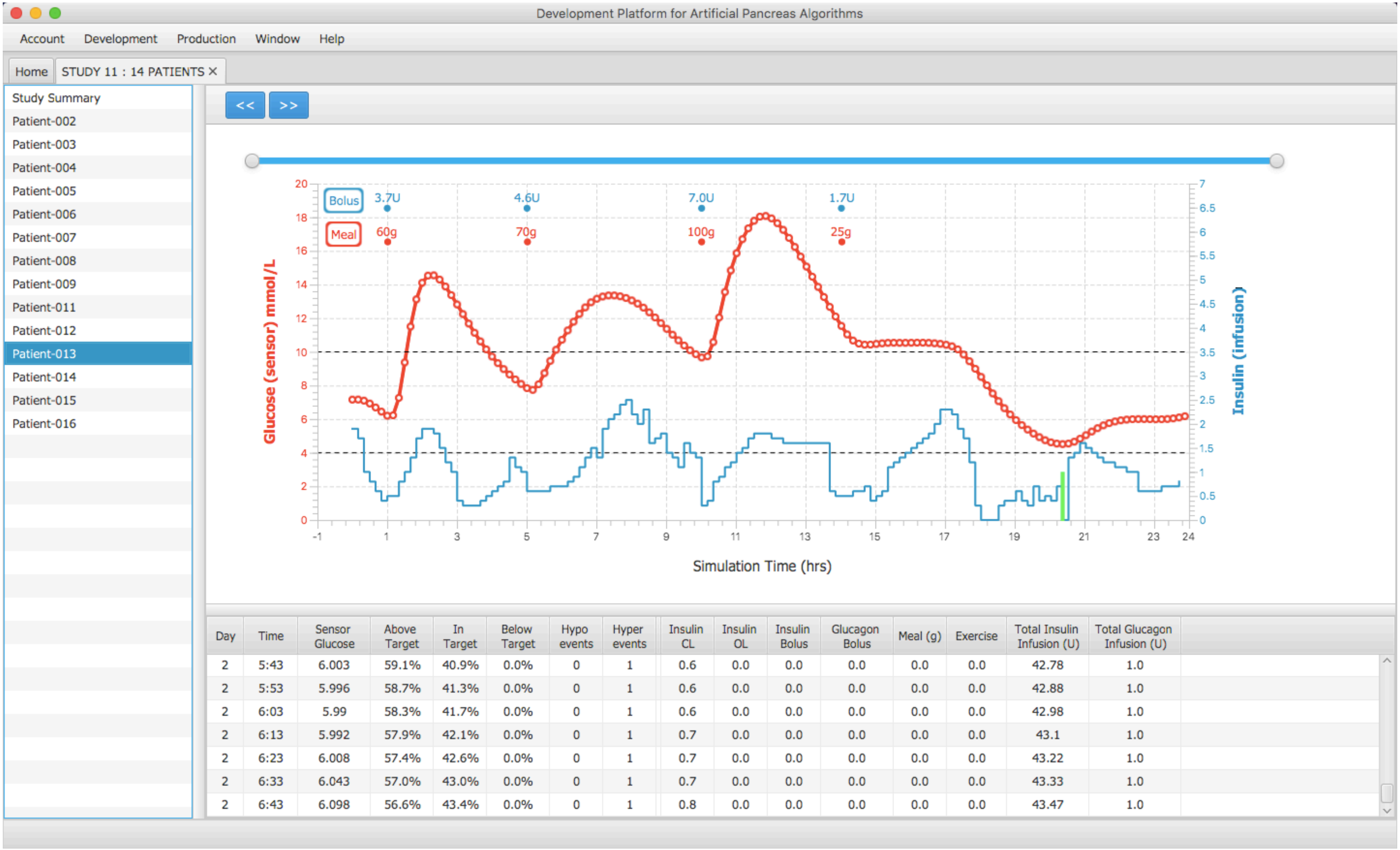

Day	Time	Sensor Glucose	Above Target	In Target	Below Target	Hypo events	Hyper events	Insulin CL	Insulin OL	Insulin Bolus	Glucagon Bolus	Meal (g)	Exercise	Total Insulin Infusion (U)	Total Glucagon Infusion (U)
2	5:43	6.003	59.1%	40.9%	0.0%	0	1	0.6	0.0	0.0	0.0	0.0	0.0	42.78	1.0
2	5:53	5.996	58.7%	41.3%	0.0%	0	1	0.6	0.0	0.0	0.0	0.0	0.0	42.88	1.0
2	6:03	5.99	58.3%	41.7%	0.0%	0	1	0.6	0.0	0.0	0.0	0.0	0.0	42.98	1.0
2	6:13	5.992	57.9%	42.1%	0.0%	0	1	0.7	0.0	0.0	0.0	0.0	0.0	43.1	1.0
2	6:23	6.008	57.4%	42.6%	0.0%	0	1	0.7	0.0	0.0	0.0	0.0	0.0	43.22	1.0
2	6:33	6.043	57.0%	43.0%	0.0%	0	1	0.7	0.0	0.0	0.0	0.0	0.0	43.33	1.0
2	6:43	6.098	56.6%	43.4%	0.0%	0	1	0.8	0.0	0.0	0.0	0.0	0.0	43.47	1.0

Figure S10: Virtual Patient-13 response graph during the dual-hormone treatment.

Table S1. Baseline characteristics of study participants

	CLASS03 (n = 29)		Simulation (n = 14)	
**Characteristic**	**Mean (SD)**	**Range**	**Mean (SD)**	**Range**
Age (years)	33 (18)	12 – 69	47 (12)	22 – 67
Body mass index (kg/m2)	24.3 (4.4)	16.9 – 34.2	26.3 (4.3)	19.2 – 34.5
HbA1c (%)	7.7 (1.0)	5.2 – 9.9	7.9 (0.6)	6.5 – 9.2
HbA1c (mmol/mol)	60.3 (10.5)	33.3 – 84.7	62.9 (7.1)	47.5 – 77
Duration of diabetes (years)	16 (11)	2 – 39	28 (14)	8 – 55
Total daily insulin dose (U)			43 (10.2)	23.2 – 62
Total daily insulin dose (U/kg)	0.70 (0.16)	0.46 – 1.08	0.99 (0.38)	0.39 – 1.73

Table S2. Comparisons of dual-hormone artificial pancreas, single-hormone artificial pancreas, and conventional insulin pump therapy for the overall Simulation study period (0800 h to 0800 h)

Outcome	Conventional insulin pump therapy (n=14)	Single-hormone artificial pancreas (n=14)	Dual-hormone artificial pancreas (n=14)	Paired difference, P value¥	Paired difference, P value‡	Paired difference, P value£
Time spent at glucose levels (%):						
In target*	52% (25)	63% (20)	64% (25)	11% (10), 0.009	12% (11), 0.001	1% (11), 0.78
4.0–10.0 mmol/L	63% (19)	74% (17)	74% (22)	11% (11), 0.008	11% (13), 0.007	1% (10), 0.8
< 4.0 mmol/L	9.1% (11.5), 1.7% (0.0-17.5)	4.3% (5.3), 1.7% (0.0-7.9)	1.2% (2.9), 0.0% (0.0-0.0)	-4.8% (8.0), 0.039	-7.9% (10.1), 0.014	-3.1% (4.6), 0.03
< 3.5 mmol/L	5.9% (8.5), 0.0% (0.0-11.0)	1.9% (3.0), 0.0% (0.0-3.3)	0.5% (1.4), 0.0% (0.0-0.0)	-3.9% (6.5), 0.037	-5.3% (0.0), 0.025	-1.4% (2.6), 0.07
< 3.3 mmol/L	4.7% (6.7), 0.0% (0.0-8.7)	1.4% (2.3), 0.0% (0.0-2.1)	0.3% (0.9), 0.0% (0.0-0.0)	-3.3% (5.1), 0.05	-4.4% (0.0), 0.02	-1% (1.9), 0.06
> 8.0 mmol/L	45% (33)	40% (25)	42% (26)	-4% (11), 0.1	-3% (11), 0.38	2% (9), 0.53
> 10.0 mmol/L	28% (26)	22% (19)	24% (23)	-6% (12), 0.045	-3% (11), 0.3	2% (7), 0.27
Mean glucose (mmol/L)	8.0 (2.5)	7.9 (1.7)	8.3 (1.9)	-0.1 (1.2), 0.48	0.2 (1.0), 0.4	0.3 (0.4), 0.02
SD of glucose (mmol/L)	2.5 (0.8)	2.4 (1.1)	2.3 (1.1)	-0.1 (0.7), 0.12	-0.1 (0.75), 0.58	0.0 (0.6), 0.91
Insulin delivery (U/kg)	1.1 (0.38)	1.0 (0.4)	1.1 (0.44)	-0.1 (0.1), 0.1	0.0 (0.1), 0.94	0.1 (0.0), 0.0002
Glucagon delivery (mg)	-	-	0.03 (0.00-0.12)	-	-	-

Data are mean (SD) or median (IQR), unless otherwise indicated. *Primary outcome, defined as 4·0–10·0 mmol/L for 2 h postprandially and 4·0–8·0 mmol/L otherwise. ¥Single-hormone system versus conventional insulin pump therapy, paired difference is single-hormone system minus conventional insulin pump therapy. ‡Dual-hormone system versus conventional insulin pump therapy, paired difference is dual-hormone system minus conventional insulin pump therapy. £Dual-hormone system versus single-hormone system, paired difference is dual-hormone system minus single-hormone system. P value less than 0.0167 is regarded as significant.

Table S3. Overnight (2300 h to 0800 h) comparisons between Simulated and Real Experiments using clinical data collected in the CLASS03 randomized trial involving the dual-hormone artificial pancreas, single-hormone artificial pancreas, and conventional insulin pump therapy arms

Outcome	Conventional insulin pump therapy		Single-hormone artificial pancreas		Dual-hormone artificial pancreas	
	CLASS03 (n=29)	Simulations (n=14)	CLASS03 (n=30)	Simulations (n=14)	CLASS03 (n=29)	Simulations (n=14)
Time spent at glucose levels (%):						
In target*	46% (35)	47% (27)	70% (27)	62% (23)	65% (30)	63% (27)
4.0–10.0 mmol/L	59% (37)	60% (21)	86% (15)	73% (18)	84% (20)	74% (23)
< 4.0 mmol/L	0.0% (0.0-21.8)	0.0% (0.0-21.0)	0.0% (0.0-3.1)	0.0% (0.0-9.4)	0.0% (0.0-0.0)	0.0% (0.0-0.0)
< 3.5 mmol/L	0.0% (0.0-12.6)	0.0% (0.0-13.2)	0.0% (0.0-0.0)	0.0% (0.0-4.0)	0.0% (0.0-0.0)	0.0% (0.0-0.0)
< 3.3 mmol/L	0.0% (0.0-7.9)	0.0% (0.0-10.5)	0.0% (0.0-0.0)	0.0% (0.0-2.5)	0.0% (0.0-0.0)	0.0% (0.0-0.0)
> 8.0 mmol/L	41% (42)	48% (36)	27% (28)	42% (29)	33% (30)	42% (29)
> 10.0 mmol/L	28% (42)	30% (29)	10% (16)	22% (21)	14% (21)	24% (24)
Mean glucose (mmol/L)	8.0 (3.3)	8.2 (2.8)	7.0 (1.5)	8.0 (1.9)	7.5 (1.9)	8.3 (2.0)
SD of glucose (mmol/L)	1.4 (1.0)	2.4 (0.8)	1.6 (1.0)	2.3 (1.1)	1.6 (1.1)	2.3 (1.1)

Data are mean (SD) or median (IQR), unless otherwise indicated. *Primary outcome, defined as 4·0–10·0 mmol/L for 2 h postprandially and 4·0–8·0 mmol/L otherwise.
