# Supplementary material for: Development platform for artificial pancreas algorithms
Source: PLoS One. 2020 Dec 17;15(12):e0243139. doi: 10.1371/journal.pone.0243139 (PMC7746189; doi:10.1371/journal.pone.0243139)
Supplement: Attachment — Submitted filename: Response_to_reviewers.docx [file pone.0243139.s002.docx]

**Reviewer 1:**

We sincerely thank the reviewer for their time in reading the manuscript and recommending it for publication. We are delighted that the reviewer has enjoyed reviewing the work.

**Reviewer 2:**

We thank the reviewer for taking the time to read and review the manuscript. We agree with the reviewer that more clinical trials on patients with type 1 diabetes are needed to better understand glucose metabolism. Data from future clinical trials in the field will continue to improve our patient models and provide better testing frameworks for algorithms. Nonetheless, the community of AP algorithm developers can still greatly benefit from the currently existing clinical data and models provided by a platform such as the one we are presenting to test and improve algorithms.

**Reviewer 3:**

We thank the reviewer for their time and effort in reviewing the manuscript and are delighted that the reviewer found the topic of high importance. We are confident of the correctness of the mathematical and statistical models.

Concern:

I would recommend not to use the term “patient” for users of AP technology throughout the manuscript.

Response:

We have reworded most of the phrases that referred to users of AP technology as “patients”. We also replaced the term with “participants” / “subjects” / “users” / “humans” throughout the manuscript wherever it seemed appropriate.

Concern:

Line 90: “algorithms must prove effective clinically in..” please check the grammar.

Response:

We thank the reviewer for pointing this out. We have rephrased the sentence to fix the grammar mistake.
